# Investigating Crimean–Congo haemorrhagic fever virus seropositivity in camels and human behavioural risks in an abattoir in Nigeria

**DOI:** 10.1017/S0950268824000189

**Published:** 2024-02-01

**Authors:** Andrew Musa Adamu, Anyebe Bernard Onoja, Victoria Ehinor Ugbodu, Reuben Sylvester Bala, Meshach Maina, Usman Shehu Salisu, Shedrach Benjamin Pewan, Emmanuel David, Arhyel Malgwi, Cornelius Adamu, Abdulrahman Adeiza, Megan Herbert, Paul Horwood, Oyelola Adegboye

**Affiliations:** 1 Australian Institute of Tropical Health and Medicine, James Cook University, Townsville, Australia; 2 College of Public Health, Medical and Veterinary Sciences, James Cook University, Townsville, Australia; 3 Department of Veterinary Public Health and Preventive Medicine, University of Abuja, Abuja, Nigeria; 4 Department of Virology, University of Ibadan, Ibadan, Nigeria; 5 Technical Services Division, Animal Care Services Ltd., Kano, Nigeria; 6 Department of Veterinary Microbiology, University of Maiduguri, Borno, Nigeria; 7 Department of Animal Science, Federal University Dutsin-Ma, Katsina State, Nigeria; 8 National Veterinary Research Institute, Vom, Plateau State, Nigeria; 9 Nigerian Field and Laboratory Training Programme, Abuja, Nigeria; 10 One Health (Man-Imal) Nantes College of Veterinary Medicine, Food Science and Engineering, University of Nantes, Nantes, France; 11 Menzies School of Health Research, Charles Darwin University, Darwin, Northern Territory Australia

**Keywords:** behavioural risks, camels, CCHFV, mass gatherings, Nigeria, serology

## Abstract

Crimean–Congo haemorrhagic fever virus (CCHFV) is an emerging viral pathogen with pandemic potential that is often misdiagnosed. Case fatality in low-resource settings could be up to 40% due to close contact between animals and humans. A two-year cross-sectional study was conducted in Fagge abattoir, Kano State, Nigeria, to estimate the seropositivity of CCHFV in camels using a commercial multi-species competitive enzyme-linked immunosorbent assay (ELISA). A closed-ended questionnaire was administered to the abattoir workers to assess their awareness, mitigation, and behavioural practices associated with CCHF. Of the 184 camels tested, 179 (97%) were seropositive for CCHFV (95% confidence interval (CI): 93.77, 99.11). The median (interquartile range (IQR)) age of respondents was 41 (35–52), with 62% having no education. Respondents had little knowledge about CCHFV and the concept of zoonotic disease. In this study, the high estimated prevalence of antibodies to CCHFV in camels highlights the heightened risk of transmission of CCHFV in Nigeria. Similarly, a concerning lack of knowledge and inadequate preventive practices, alongside a prevalence of high-risk behaviours associated with CCHF among abattoir workers, were noted in this study. Thus, there is an urgent need for comprehensive public health education and collaborative One Health strategies to avert the threats of spillover events.

## Outline of key results and their importance

- For the first time, high seropositivity of Crimean–Congo haemorrhagic fever virus (CCHV) was reported in Nigerian dromedaries brought for slaughter.
- A known competent vector of CCHV – *Hyalomma marginatum* (*H. marginatum*) *rufipes* – was morphologically identified.
- The study reported poor use of personal protective equipment (PPE) and limited practice of infection prevention and control measures by the abattoir workers, which may likely result in spillover.

## Introduction

The World Health Organization (WHO) defines mass gatherings as events attended by a sufficient number of people to strain the planning and response resources of the host community [1]. These events pose significant health risks when outbreaks of infectious diseases with pandemic potential occur [2, 3]. A big challenge in managing outbreaks during mass gathering events is the difficulty in detecting suspected cases, clusters, and sources of infection to implement effective preventive measures [2]. The past two decades have witnessed influenza and coronaviruses associated with respiratory infections during mass gathering events [4]. Surprisingly, no outbreaks of Middle East respiratory syndrome coronavirus (MERS-CoV) or severe acute respiratory syndrome coronavirus-1 (SARS-CoV-1) were reported during Hajj, one of the most well-studied annual global religious mass gatherings [5]. However, recent events such as the Olympic Games and certain religious gatherings were cancelled or postponed due to the severe acute respiratory syndrome coronavirus-2 (SARS-CoV-2) [3].

Crimean–Congo haemorrhagic fever (CCHF) is a tick-borne zoonotic viral disease characterized by systemic haemorrhagic symptoms in severe human cases, but it is asymptomatic in animals [6]. CCHF is caused by the CCHF virus (CCHFV), a member of the genus *Orthonairovirus* and family Nairoviridae [7]. Human infections arise from tick bites or direct contact with infected animals or animal products and present with symptoms ranging from fever, headaches, nausea, and myalgia to severe systemic haemorrhagic fever, with a case fatality rate of up to 40% [8]. The virus circulates in an enzootic tick–vertebrate–tick cycle involving various domestic animals and wildlife. Humans are typically considered dead-end hosts [6], but human–human transmission can occur from close contact with blood and bodily fluids from infected persons. Individuals at high risk of occupational exposure to CCHFV include animal and human health workers, pastoralists, and abattoir workers [9]. In abattoirs, people from different backgrounds, such as animal health workers, merchants, and butchers, congregate to process meat from various animals. CCHFV spillover events occur through interactions between humans, animals, ticks, and the environment. A multistage analysis and cohesive framework developed for viral haemorrhagic fevers with pandemic potential in Africa highlighted CCHF as a substantial risk with the potential to cause outbreaks, epidemics, and pandemics comparable to Marburg virus disease, Ebola virus disease, and Lassa fever [10]. A predictive model identified Central, Eastern, Southern, and West African regions as conducive to CCHF circulation [11].

Camels are an important multipurpose livestock species of economic importance in Africa and the Middle East. They are one of the most drought-resilient mammalian species, adapted to arid and semi-arid ecosystems [12, 13]. Global warming has intensified the risk of camels’ involvement in the complex transmission of emerging and re-emerging diseases. Interactions of camels with other livestock at water points during scarcity predispose them to exotic diseases [13]. Previous studies have reported the presence of CCHFV in ruminants and humans in Nigeria [9, 14, 22]. However, there is no report on the presence of CCHFV in camels despite their increased role in the food chain across Africa and the Middle East. Therefore, we conducted a two-part cross-sectional study to assess CCHFV seropositivity in camels and explore associated human behavioural risks among abattoir workers in Fagge abattoir, Nigeria.

## Materials and methods

### Study area

This study was conducted in Fagge abattoir, Kano, in the north-western region of Nigeria (latitude: 12°0′7.8444’N and longitude: 8° 35′ 31.0416″ E). Kano State shares borders with Katsina, Jigawa, Bauchi, and Kaduna states. Its climate is tropical, dry, and wet (Aw Köppen classification) with Sudan savannah and Sahel savannah vegetation [15]. Rainfall occurs mainly between June and September, with temperatures ranging from 26 °C to 33 °C [16]. The Fagge abattoir, established in 1963, serves as a central hub for slaughtering cattle, camels, and small ruminants, with variable daily averages influenced by factors such as religious festivals (such as Eid al-Adha), drawing livestock from various sources, including rural areas, international markets, neighbouring states, and West Africa. Approximately 80,000 individuals are engaged in the meat processing value chain within this abattoir, encompassing livestock merchants, hide and skin dealers, pastoralists, and meat sellers [40].

### Study design/sampling frame

This was a cross-sectional study conducted between June 2021 and May 2022. One hundred and eighty-four one-humped camels (dromedaries) presented for slaughter at the Fagge abattoir were randomly selected (a total of 23 every fortnight of the study period). Age and sex were recorded for each animal. An attendant restrained each camel in a crouching position, and 5 mL of blood was collected via the jugular vein using an 18-G needle and a 10-mL syringe. The blood was transferred into plain clotting tubes and kept on ice packs in an insulated box for transport to the Animal Care Laboratory, Kano. Samples were centrifuged at 10,000 g for 5 min to allow for proper separation of serum from the clotted blood. Using a sterile pasture pipette, the serum was transferred into appropriately labelled 2-mL cryovial tubes and stored at −20 °C before transportation to the Virology Laboratory, University of Ibadan, for serological analysis.

### Body scoring

Camels were scored into one of four classes of body condition: type 1 – very thin (without hump), type 2 – thin, type 3 – good, and finally type 4 – very fat. Scoring was performed using measurements of accumulated hump fats and the circumference of the thigh, which butchers use as a reference [17].

### Ticks

The entire bodies of camels were routinely checked to avoid bias and ensure that representative specimens were collected rather than only checking known predilection areas. Ticks were removed using forceps and placed in a tube containing 70% ethanol. Ticks were transported to the Department of Veterinary Parasitology and Entomology Laboratory (Ahmadu Bello University, Zaria) for morphological identification of species as previously described [18].

### Serological assay for CCHFV

The blood samples were analysed for CCHFV antibodies using an established enzyme-linked immunosorbent assay (ELISA) (ID Screen® CCFH Double-Antigen Multi-Species ELISA Kit) according to the manufacturer’s instructions (IDvet Innovation Diagnostic, Grenoble, France). This diagnostic kit is designed to detect specific antibodies against the nucleoprotein (NP) of CCHFV in the sera of susceptible animal species. All laboratory procedures were carried out at 21 °C (±5 °C) using a similar protocol described by Dzikwi-Emennaa et al. [9]. The optical density (OD) of each microplate was read at 450 nm using a plate reader (Thermo Scientific™ Multiskan™, Waltham, MA, USA). Percentage seropositivity (S/P) for each sample was calculated by dividing the OD value of each sample (OD_S_) by the OD of the positive control (OD_PC_), multiplied by one hundred. Samples with S/P values greater than 30% were considered positive, while those less than or equal to 30% were considered negative.

### Questionnaire administration

A closed-ended questionnaire was randomly administered to abattoir workers at Fagge. This questionnaire was pre-tested in a pilot sample of abattoir workers to ascertain face and content validity. All questions were written in English but were administered through oral interviews in Hausa, the predominantly spoken language in Kano State. The survey questionnaire was administered face-to-face by five para-veterinarians who were proficient in both English and Hausa and were instructed in the survey implementation by a veterinarian (RSB). The respondents were informed about the aims of the study, and verbal consent was obtained from each respondent and recorded on the paper-based questionnaire. Respondents’ voluntary participation was assured in accordance with the Helsinki Declaration [19].

The questionnaire included socio-demographic variables such as age, sex, tribe, marital status, occupation, duration of employment in the abattoir, and level of education. Participants’ knowledge/awareness of CCHF was assessed to determine their understanding of how the disease can affect camels, its zoonotic significance, and how to recognize clinical signs in camels based on their observations and experience working in the abattoir. Additionally, respondents were asked about risk activities such as handling ticks with bare hands, consuming raw meat, handling sick animals, assisting deliveries, and purchasing blood from the abattoir. Respondents were further asked about the use of personal protective equipment (PPE) and infection, prevention, and control (IPC) practices while working in the abattoir, such as the use of gloves, hand washing or disinfectant use, bathing after work, the use of protective shoes, and the use of protective eye shields or masks.

### Statistical analysis

Data analyses were performed in R version 4.0.1, and all tests were based on a 5% significance level. The distribution of camel characteristics, seropositivity, and human behavioural data was described by frequencies and percentages for categorical variables and median and interquartile ranges (IQRs) for numerical attributes. The proportion of seropositivity to CCHFV in camels was computed as the number of seropositive camels divided by the total number of camels tested. To determine the uncertainty around this proportion estimate, Clopper–Pearson’s 95% confidence interval (CI) was calculated. Fisher’s exact test and Wilcoxon’s rank-sum test were utilized to examine the variation in seropositivity across different camel and human characteristics. Furthermore, for potential risk factors associated with CCHFV seropositivity in camels, we employed Firth’s logistic regression to handle small-sample bias and address issues arising from the separation or quasi-separation of data [20, 21].

## Results

### Camel characteristics and risk factors

Overall, 179 of 184 camels (97%) were seropositive for CCHFV, with an estimate of uncertainty (95% CI: 93.77, 99.11). Among 146 female camels, 144 were seropositive, and two were seronegative. Of the 38 male camels, 35 were seropositive, and three were seronegative. The median age of the camels was ten years (IQR: 8–14). Among camels aged less than 5 years, which were considered young, three out of five were seropositive, while old camels aged greater than 5 years had 176 out of 179 seropositivity. All camels with a body score of 2 or 4, and 93% of camels with a body score of 2, had antibodies to CCHFV (Table 1). The potential intrinsic risk factors for CCHFV transmission in camels were analysed using Firth’s logistic regression. Male camels had a significantly lower risk of CCHFV transmission than female camels (adjusted odds ratio (aOR) = 0.09, 95% CI: 0.01 to 0.79). Similarly, camels within the categorized age greater than 5 years were 72.22 times more likely to be infected with CCHV compared with young camels (95% CI: 5.59 to 1831.14) (Table 2).

**Table 1.** Demographic and seroprevalence of CCHV among camels slaughtered in Kano abattoir, Nigeria

Variable	Total	Negative, n (%)	Positive, n (%)	*p*-value^a^	% (95% CI)^b^
Overall	184	5 (2.7)	179 (97)		97.28 (93.77,99.11)
Sex, n (%)				0.06	
Female	146 (79)	2 (1.1)	144 (78)		98.63 (95.14, 99.83)
Male	38 (21)	3 (1.6)	35 (19)		5.26 (0.64, 17.75)
Age, median (IQR)	10 (8–14)	10 (4–12)	10 (8–14)	0.22	
Age group				0.006	
< 5 years	5 (2.7)	2 (1.1)	3 (1.6)		60.00 (14.66, 94.73)
> 5 years	179 (97)	3 (1.6)	176 (96)		98.32 (95.18, 99.65)
Body score, n (%)				0.027	
2	2 (1.1)	0 (0)	2 (1.1)		100.00 (15.81, 100.00)
3	74 (40)	5 (2.7)	69 (38)		93.24 (84.93, 97.77)
4	108 (59)	0 (0)	108 (59)		100.00 (96.64, 100.00)

a Fisher’s exact test; Wilcoxon rank-sum test.

b Clooper–Pearson’s 95% confidence interval.

**Table 2.** Potential intrinsic risk factors for CCHFV transmission in camels slaughtered in Kano abattoir, Nigeria

	aOR (95% CI)	*p*-value
Gender		
Male	0.09 (0.01, 0.79)	0.0339^a^
Female		
Age		
< 5 years	ref	
> 5 years	72.22 (5.59, 1831.14)	0.001^a^

Abbreviations: aOR, adjusted odds ratio.

a *p*-value <0.05.

### Abattoir workers’ characteristics and human behavioural risk assessment

Of the 189 respondents, 184 (97%) were male, while five (2.6%) were female. The median age of respondents was 41 years (35–52). The marital status shows 145 (77%) were married, 38 (20%) were single, and only 1 (0.5%) was divorced. Based on occupation, meat sellers constituted the highest number of respondents (n = 86; 46%), followed by butchers (n = 68; 36%). A high proportion of respondents (n = 117, 62%) indicated they were not educated (Table 3). Responses regarding knowledge of CCHF showed that 152 (80%) respondents had not heard of CCHFV, while 146 (77.4%) were not aware that CCHFV could affect camels, and 96 (51.3%) were not aware CCHFV was a zoonotic disease. Most respondents had worked for a median period of 20 years (IQR: 12–30) in the abattoir (Table 4).

**Table 3.** Respondents’ socio-demographic profile in Fagge abattoir, Kano, Nigeria

Characteristic	N = 189
Age, median (IQR)	41 (35–52)
Gender, n (%)	
Female	5 (2.6)
Male	184 (97)
Tribe, n (%)	
Hausa	182 (96)
Yoruba	2 (1.1)
Marital status, n (%)	
Divorced	1 (0.5)
Married	145 (77)
Single	38 (20)
Occupation, n (%)	
Animal health worker	17 (9.0)
Businessman	1 (0.5)
Butcher	68 (36)
Butcher, businessman	6 (3.2)
Meat seller	86 (46)
Education, n (%)	
None	117 (62)
Others	3 (1.6)
Primary	17 (9.0)
Secondary	33 (17)
Tertiary	14 (7.4)

**Table 4.** Respondent’s awareness/knowledge of CCHF in Fagge abattoir, Kano, Nigeria

Characteristic	N = 189
Have you ever heard about CCHFV?	
Not recorded	5 (2.6)
No	152 (80)
Yes	32 (17)
Source of CCHFV information?	
	156 (83)
Community meetings	2 (1.1)
Friends	2 (1.1)
Radio	11 (5.8)
School	1 (0.5)
Veterinary/health workers	17 (9.0)
Do you think CCHFV can affect camels?	
Not recorded	5 (2.6)
No	146 (77.4)
Yes	38 (20)
Is CCHFV transmissible from animals to humans (zoonosis)?	
No	96 (51.3)
Yes	88 (47)
How long have you been working in the abattoir? Median (IQR)	20 (12–30)
Is CCHFV routinely detected (endemic) in an abattoir?	
No	131 (69.8)
Yes	53 (28)

Respondents engaged in several behavioural risk activities that could predispose them to CCHFV infection. One hundred and thirty-four respondents affirmed having slaughtered sick animals (73%), and 95 (52%) handled ticks with their bare hands. Furthermore, 138 (75%) consumed raw meat when working in the abattoir, and 96 (51%) participated in assisted deliveries of pregnant animals, with 35% affirming that people buy blood from the abattoir (Table 5).

**Table 5.** Respondents who responded to behavioural risk practices associated with CCHF in Fagge abattoir, Nigeria

Characteristic	N (%)
Engaged in slaughter of sick animal	134 (73)
Handling of ticks with bare hands	95 (52)
Separated healthy animals from infected ones before slaughter	37 (20)
Performed assisted delivery in pregnant animals	94 (51)
Knowledge on getting infected from the consumption of raw meat	41 (22)
Engage in raw meat consumption	138 (75)
Purchase of slaughtered animal blood from the abattoir	64(35)

**Figure 1.**
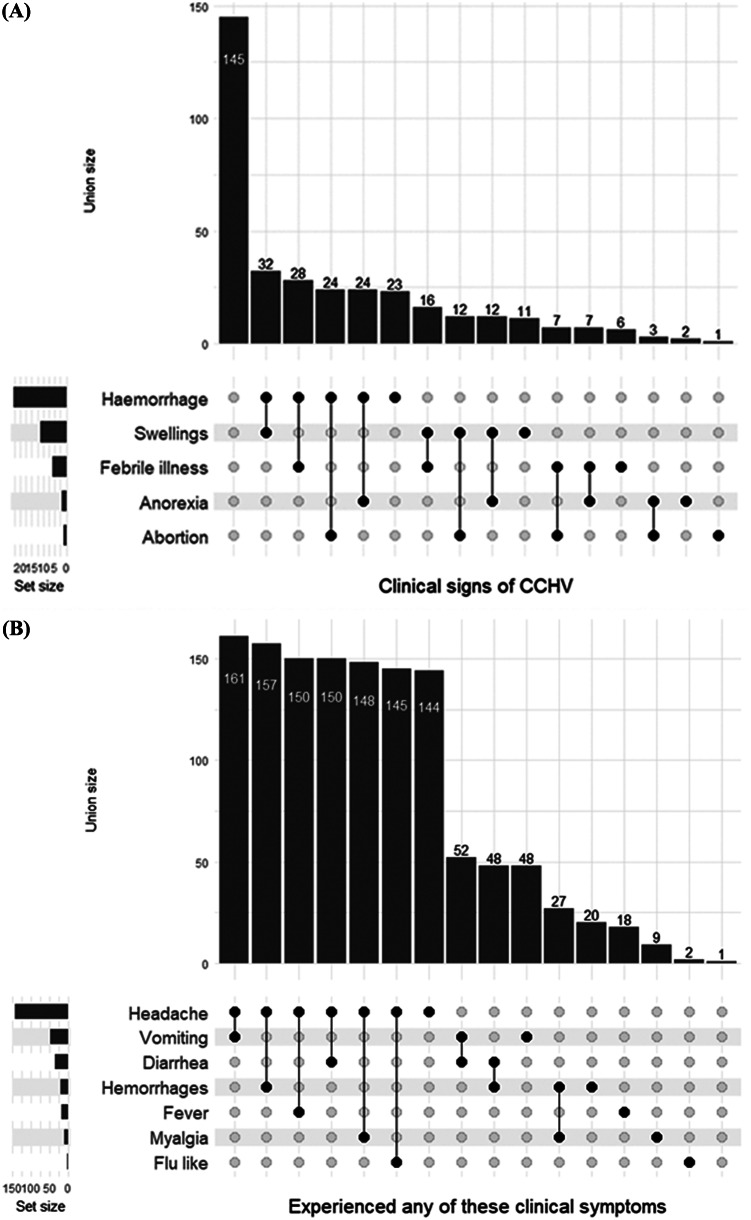
(a) Clinical signs of CCHV observed in camels and (b) clinical symptoms experienced by respondents working in Fagge abattoir, Kano, Nigeria.

**Figure 2.**
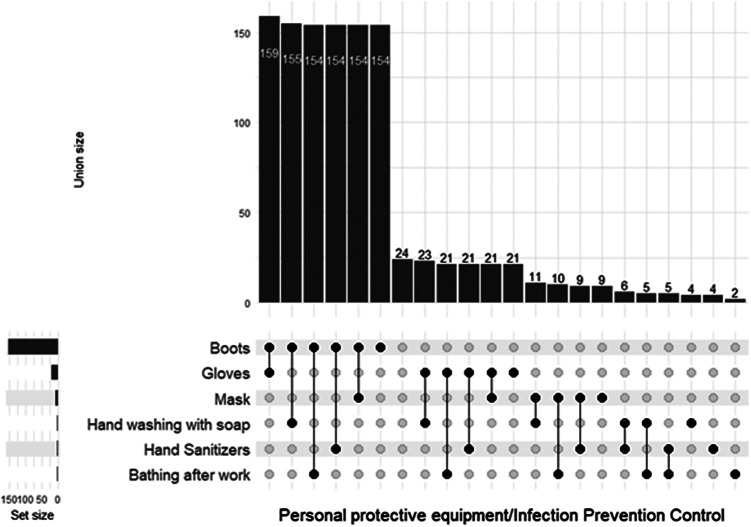
Mitigation practices (personal protective equipment and infection prevention and control) employed by abattoir workers in Fagge abattoir, Kano, Nigeria.

In this study, abattoir workers were assessed for their awareness and knowledge of clinical signs associated with CCHFV. The union size of the data member is shown in Figure 1a. Participants were mostly aware of the following clinical signs of CCHFV: haemorrhage, swelling, and febrile illness. Clinical symptoms of respondents that could be associated with CCHFV include headache, the most common symptom of CCHFV, followed by vomiting, diarrhoea, haemorrhages, and fever, respectively (Figure 1b). The use of PPE and infection prevention and control measures play a vital role in mitigating the spread or transmission of CCHFV and other infectious diseases. The set size in Figure 2 indicates that boots were the major form of PPE, while infection prevention and control practices were rarely observed.

### Morphological identification of ticks

A stereomicroscope ST-6-1331 (Steindorff, Hicksville, NY, USA) was used for viewing and identification of the collected ticks. Overall, 75 male and female adult ticks were collected and identified as *Hyalomma marginatum (H. marginatum) rufipes* (45), *Amblyomma variegatum* (21), and *Hyalomma dromedarii* (9).

## Discussion

This study estimated camel exposure to CCHFV in Fagge abattoir in Kano State, Nigeria. Overall, seropositivity of 98% was recorded among camels brought for slaughter during the study period. Previous seroprevalence estimates among ruminants (cattle, sheep, and goats) in Nigeria range from 2% to 30% [9, 22], but this is the first study to report on CCHFV in camels in Nigeria. Globally, there are few reports of CCHFV in camels, and the rates of seropositivity vary widely: 5.29% in Iran [23], 14% in Egypt [24], 21% in Sudan [25], 47.5% in the Niger Republic [26], 81% in Mauritania [27], and 84% in the United Arab Emirates [28].

Despite the resilience of camels to drought, their nutrition and health can be significantly altered by the depletion of grazing land and increases in extreme weather events, predisposing them to emerging and re-emerging infectious diseases. A vast majority of camels in Nigeria are raised extensively, where they are exposed to extrinsic environmental factors suitable for vector proliferation. Recent studies have highlighted a predisposition of camels to zoonotic pathogens such as influenza A virus [29], MERS-CoV [30], Rift Valley fever virus [31], and hepatitis E virus [32]. CCHFV is an arboviral infection that can be categorized under acute undifferentiated febrile illness (AFI) in humans with unspecified clinical manifestations ranging from fever, headache, and malaise [33]. Clinical symptoms can be mild or progressive to life-threatening. However, in resource-limited settings, clinical diagnoses and testing to verify the aetiologies of AFIs are limited, and most are treated with antibiotics [34]. CCHF can be misdiagnosed without obvious features and low levels of disease surveillance [7, 35].

With thousands of people engaged in raw meat processing in Fagge abattoir, the likelihood of exposure to pathogens during mass gatherings at abattoirs, religious gatherings, or sporting events could be high and increase the potential for spillover events. Slaughtering sick animals, handling ticks with bare hands, consuming raw meat, and participating in assisted deliveries were behavioural risk activities practised by respondents. A recent CCHF outbreak in Afghanistan was linked to people working in the livestock industry and health settings who failed to use PPE and observed minimal infection prevention and control practices [36]. Public health education should be prioritized among abattoir workers to mitigate behavioural risk practices and increase awareness of CCHF.

The *Hyalomma* and *Amblyomma* tick species collected and morphologically identified in our study are known to be the principal vectors in the transmission of zoonotic CCHFV [37]. Reports have shown that *H. marginatum rufipes* naturally harbours several different genotypes of CCHFV and, in some regions, is postulated to play a significant role in maintaining viral endemicity via transovarial or transstadial transmission [38, 39]. Previous studies have linked autochthonous CCHF cases through epidemiological surveys or CCHFV isolation in ticks to indicate local CCHFV covert circulation [38]. Similarly, *Amblyomma variegatum*, present in both Africa and Europe, is reportedly capable of transmitting CCHFV [7]. The presence of these ticks in camels in close proximity to humans in the abattoir could result in a CCHFV spillover event.

Our study is not without limitations. First, we could only identify competent vectors of CCHFV morphologically but could not detect CCHFV ribonucleic acid (RNA) in the ticks, which could buttress our findings. Second, although we used commercially available kits for our serology, we cannot rule out the possibility of antigenic cross-reactions with other nairoviruses. However, the detection of antibodies in camel sera and the identification of the primary tick vector for CCHFV in camels signify a potential risk for the circulation of this virus in humans [39]. Future studies should investigate the presence of CCHFV and the force of infection in humans using longitudinal studies incorporating One Health strategies. We also observed a significant imbalance in both sex and age groups, which may impact statistical power and model sensitivity due to larger group disparities. This observation aligns with the typical scenario where older female camels are more frequently presented for slaughter. However, the statistical techniques employed have effectively served the analytical objectives of this study.

In conclusion, this study has highlighted the presence of CCHFV antibodies in camels, human behavioural risk factors, and morphological identification of CCHFV-competent tick vectors. To avert the threat of CCHFV transmission in abattoirs and other similar settings, a risk assessment framework should be implemented to reduce the risk of human transmission. The absence of CCHF vaccines further calls for heightened public health education, strategic surveillance, appropriate testing, and epidemiological evaluation of emerging infectious diseases.

### Ethical considerations

This work was approved by the Government of Kano State under the ethical clearance of the Ministry of Agriculture and Natural Resources dated 22 June 2021 and referenced in REF.VET/80/EC.21. Verbal consent was obtained from all the major stakeholders in the abattoir before the commencement of sampling and questionnaire administration.

## Data Availability

Data availability will be available upon reasonable request.
